# Reviewer List 2023

**DOI:** 10.1093/af/vfae001

**Published:** 2024-02-14

The Section Editors and the Editor-In-Chief of *Animal Frontiers* like to thank the many scientists who have contributed their time and talents reviewing manuscripts for the journal. Reviewing for the journal is critical to ensuring that the best science is published. The expertise of these individuals is invaluable to the journal, the animal science community and to those that depend on the high-quality science that is published.

## Top reviewers (3+ reviews completed)

DiGiacomo, Kristy

Gortazar, Christian

Gross, Josef

Zinn, Steven

## Reviewers

Allen, Benjamin

Apollonio, Marco

Argüello, Anastasio

Babarinde, Samuel A.

Bacenetti, Jacopo

Baes, Christine

Barragan, Wilson

Barroso, Patricia

Baumrucker, Craig

Behrouzi, Amir

Blum, Jürg

Boler, Dustin

Brien, Forbes

Bruckmaier, Rupert

Cammack, J.A.

Carrejo, Nancy

Castro, Noemí

Caton, Joel

Chehma, Abdelmadjid

Chinn, Sarah

Clarke, Iain

Colle, Michael

da Costa, Luciana

Dahl, Geoffrey E.

Daniel, Jay

Devic, Emilie

Di Stasio, Liliana

Didkovska, Anna

Downing, Jeff

Duarte, Marcio

Duff, Glenn

Dunshea, Frank

Durunna, Obioha

Elmaz, Özkam

Farmer, Chantal

Finck, Jessica

Forster, Ian

Fowler, Melinda

Fragomeni, Breno

Fynn, Richard

Galbraith, Jayson

Garmyn, Andrea

Gaughan, John

Gonzalez, John

Goodband, Robert

Gordon, Iain

Guo, Kaijun

Halmemies-Beauchet-Filleau, Anni

Hamernik, Deb

Hansen, Peter

Hodgen, Jennie

Hood, Wendy

Jiménez-Ruiz, Saúl

Kemper, Nicole

Kensinger, Ron

Kovitvadhi, Attawit

Lalander, Cecilia

Lawrence, Ty

Liu, Christine

MacNeil, Michael

Matandirotya, Newton R.

Meijer, Nathan

Meneguz, Marco

Miller, Phillip

Mishyna, Maryia

Mortimer, Sue

Mysterud, Atle

Obese, Frederick

Osburn, Wes

Owino, Victor

Power, Michael

Reed, Sarah

Rincker, Phil

Romelle Jones, Kegan

Rubio Lozano, María Salud

Rumbos, Christos I.

Rumpold, Birgitte

Sabia, Emilio

Safran, Amy

Schokker, Dirkjan

Serrano Ferron, Emmanuel

Smith, Jennifer

Soladoye, Philip

Sosnicki, Andrzej

Thomson, Jennifer

Tolleson, Douglas

Tomberlin, Jeffery K.

Tran, Hung Quang

Vishnuraj, M. R.

von Cramon-Taubadel, Noreen

Youngs, Curtis

Zhai, Chaoyu

Zhang, Peipei

Zhang, Yi

